# Liquid Patterning Using Droplet Impact on Textured Nonwetting Surfaces

**DOI:** 10.1021/acsami.5c23079

**Published:** 2026-01-20

**Authors:** Biruk Teka Gidreta, Elijah Williams, Michal Remer, Solomon Adera

**Affiliations:** 1 Energy Transport Lab (ETL), Department of Mechanical Engineering, 1259 University of Michigan, Ann Arbor, Michigan 48109-1382, United States; 2 Institute of Aeronautics and Applied Mechanics, Warsaw University of Technology, Warsaw 00-665, Poland

**Keywords:** liquid patterning, micropillars, droplet impact, nonwetting surfaces, Wenzel state

## Abstract

Controlling the shape and contact area that an impacting droplet makes with a solid substrate has significant implications in numerous industrial processes, including inkjet printing and spray cooling. Here, we report a unique approach that offers an extraordinary ability to precisely control and manipulate the contact shape of a droplet impinging on nonwetting well-structured silicon micropillars. Our experiments show that the wetted Wenzel-type contact area can take on various polygonal shapes, including square, rectangle, hexagon, octagon, and dodecagon, depending on the pillar density (diameter-to-spacing ratio), arrangement (inline versus staggered), and/or the droplet contact angle. Experiments show that inline pillars give rise to a square, rectangle, or octagon shape while staggered pillars give rise to a hexagon, dodecagon, or extended hexagon shape. Rooted in the fundamentals of contact line physics, we develope a closed form unified analytical model that accurately captures the steady-state and transient wetting morphology of the impinging droplet. Furthermore, we show that the model is applicable for analyzing entrapped bubble retraction mechanism during high-velocity droplet impact. Lastly, the outcomes of this study demonstrate the similarity of the shape of the wetted area induced by droplet impact on nonwetting surfaces with that obtained via sessile droplet evaporation on wetting surfaces. The shape selection strategy reported in this study has promising applications in facile microfabrication of lab-on-a-chip devices, polymer-based printed electronics, biomicroarrays, and droplet-based electronics thermal management.

## Graphical abstract

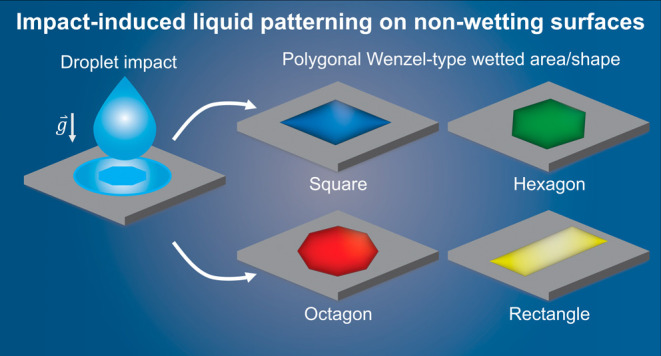

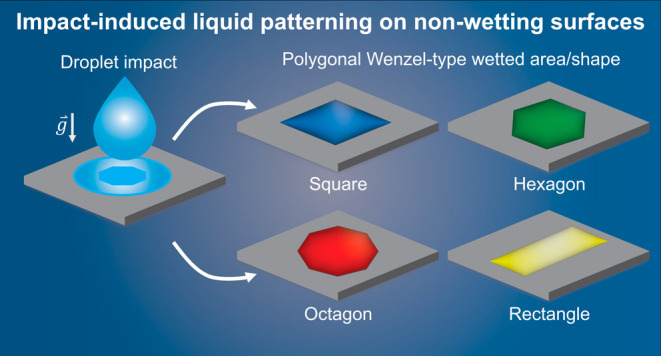

## Introduction

Droplet-substrate interaction is ubiquitous in nature and in industry.^1–4^ In nature, raindrops impact plant leaves, insect wings, and soil surfaces, influencing processes such as water harvesting, self-cleaning, and erosion. The ability to repel liquids for many animals and plants is an evolutionary adaptation for survival. For example, insects must avoid getting trapped by falling raindrops, and plants need to keep their leaves dry for efficient photosynthesis by maintaining their stomata open.^5–8^ As a result, many animal and plant surfaces are decorated with hierarchical micro/nanostructures or wax-like coatings to prevent fouling by increasing liquid-repellency.^9–13^ Similarly, in industry, droplet impact plays a critical role in applications ranging from inkjet printing and spray coating to thermal management and pesticide deposition.^14–19^ Inspired by nature, topographic and chemical heterogeneities are utilized to create engineered surfaces with desired wettability.^3,5,20–24^ Of special interest is the design of nonwetting surfaces via surface roughness, whereby the surface is decorated with hierarchical micro/nanostructures to maintain a stable air layer to minimize the droplet-surface contact. This so-called Cassie state^25,26^ contrasts with the classical Wenzel state,^27^ whereby the droplet penetrates the micro/nanostructures and wets the rough surface crevices following surface topography. Such nonwetting surfaces are desirable for many applications, including self-cleaning,^1,2,28^ anti-icing,^29–32^ and condensation.^33–35^

Considerable efforts have focused on understanding the precise shape of the solid–liquid contact area on microstructured surfaces.^36–45^ Earlier attempts to shape the droplet-surface contact area have shown that droplets in the Wenzel state can take on various shapes by manipulating the microstructure geometry on wetting surfaces. Droplets deposited on highly wetting surfaces satisfying the imbibition criteria^46–48^ were shown to be manipulated to create various polygonal shapes depending on the micropillar geometry and the equilibrium/static contact angle.^36,49^ Another prior study demonstrated the ability to tailor the droplet-surface contact area of Wenzel-type nonimbibing droplets by controlling the pillar density of the surface and the pillar arrangement.^38^ By characterizing the side and top profiles of an evaporating sessile droplet, the study also developed a universal model to predict the contact area shape. Controlling the shape of droplets on a nonwetting surface is particularly challenging since part of the contact line pins on the solid while the remaining part is suspended in an air cushion.^21,50,51^ Furthermore, the Cassie state is metastable and can easily breakdown due to numerous factors, for example, external pressure or mechanical damage on the surface.^52–58^ More recently, an active technique to shape droplets on nonwetting surfaces has been shown, whereby confinement is imposed on a droplet by an external force.^41^ For many practical applications, the droplet-solid interaction occurs via impingement of the droplet onto a solid surface, hence moving contact line. For low-velocity impact on hydrophobic surfaces, a circular contact area is formed during spreading of a droplet in a Cassie state.^59^ Faceted droplets have been shown to form temporarily on superhydrophobic surfaces during high-velocity droplet impact, in which a polygonal shape is formed during spreading before the droplet fully retracts and bounces off the surface.

When a droplet impacts a pillar array at a sufficiently high velocity, it penetrates the structure and wets the surface by displacing the air, rather than forming a completely bouncing Fakir droplet.^60–63^ The central penetrated area will be fully wetted (Wenzel state) with an entrapped air bubble at the center, whereas the spreading liquid sheet remains largely in the Cassie state. In this study, we show that various polygonal shapes, including squares, rectangles, hexagons, octagons, dodecagons, and extended hexagons, can be obtained on nonwetting microstructured surfaces via droplet impact. This is in contrast with previous works that showed liquid shape selection on wetting surfaces (via imbibition or evaporation) or by external force on nonwetting surfaces. By controlling the pillar arrangement, pillar density, and the surface tension and the resulting contact angle of the droplet, we demonstrate the ability to selectively generate the desired polygonal shape. Using high-speed image analysis, we visualized the contact line movement at a microscale. We also show that the size and shape of the entrapped air bubble depend on the pillar density and arrangement. Furthermore, we demonstrate the similarity of the liquid shapes obtained via droplet impact and via evaporation of sessile droplets on wetting surfaces. Overall, the work reported in this study is unique in that, unlike past studies, which use micro/nanotextured wetting surfaces, our surfaces are nonwetting or superhydrophobic, and the solid–liquid contact area pattern is achieved using droplet impact, instead of relying on imbibition or evaporation. Additionally, this work is distinct from previous studies since it investigates both the steady-state as well as transient wetting dynamics. Finally, in this work, the distinct liquid shapes are obtained by varying not only the pillar geometry, which was the case in previous studies, but also the contact angle on the droplet by varying the surface tension of the liquid.

## Results and Discussion

### Experiment

We fabricated well-defined silicon pillars arranged inline and in a staggered fashion using standard silicon fabrication technology, specifically contact photolithography and deep reactive ion etching (DRIE).^64,65^ The samples were hydrophobized by coating them with a ≈100 nm thick C_4_F_8_ using chemical vapor deposition in a reactive ion etching chamber. The fluorinated test samples were wet cleaned thoroughly with acetone, ethanol, isopropanol alcohol, and deionized water in preparation for the experiments. The test samples were blow-dried using compressed nitrogen gas following the wet cleaning protocol. The temperature in the laboratory was maintained at ≈20 °C for all experiments. Detailed descriptions of the sample fabrication, chemical treatment, and scanning electron microscope (SEM) images are provided in the Methods section and Supporting Information S1. In a typical experiment, a ≈10 μL droplet released from a height of 20 cm from a microsyringe impinges on the textured surface. The corresponding Reynolds number (Re) was Re ≈ 5300. The dimensionless Reynolds number, which compares inertia to viscous forces, is given by Re = ρ*ud*/η, where ρ, η, *u*, and *d* are the density, dynamic viscosity, impact velocity, and volume-averaged diameter of the droplet before impact. The Capillary number (Ca) for the falling drop, which compares inertia with surface tension and is given by Ca = η*u*/γ, where γ is the surface tension of the drop, was Ca = 0.03 for our experiments. The dimensionless Ohnesorge number, which relates viscous forces to inertial and surface tension forces, was very low at Oh ≈ 0.002 indicating viscosity effects are relatively small compared to inertia and surface tension. Lastly, the Weber number (*We*), which compares inertia with surface tension and is given by *We* = ρ*u* ^2^ *d*/γ, was *We* ≈ 150 showing the major dominance of inertial forces over viscous and surface tension forces in droplet impact dynamics and the resulting solid–liquid contact pattern (shape and size). We estimated the impact velocity (*u*) using the energy balance by converting the gravitational potential energy of the droplet into kinetic energy at impact. Hence, for a droplet released from a height *h*, the impact velocity becomes *u* = (2*gh*)^1/2^, where *g* is the gravitational acceleration (*g* = 9.81 m/s^2^). The droplet impact dynamics was captured using a high-speed camera (Phantom v1610, Vision Research) at 5,000–33,000 frames-per-second (fps). The frame rate was determined based on the data required for analysis. The details of the experimental setup and data acquisition protocol are discussed in Supporting Information S2.

### Droplet Contact Area

When a millimeter-sized droplet impinges on a solid substrate, it forms a dimple at its center, due to the pressure buildup at the base of the droplet, as shown schematically in Figure 1a. More importantly, when the surface is hydrophobic, the droplet establishes contact with the substrate (Wenzel state)^3,27^ and entraps an air bubble that has been the subject of numerous studies in the past.^66,67^ Due to the nonwettability of the surface, the spreading liquid sheet lifts off from the surface (Cassie state)^25,68^ that will lead to droplet breakup depending on the Weber number. All or part of the liquid sheet eventually recoils back and rebounds from the surface. Here we report an interesting observation wherein the wetted area can take on various polygonal shapes depending on the pillar density and their arrangement. Importantly, this impact-induced liquid patterning can also be manipulated by varying the surface tension, hence the Young contact angle. This point will be discussed in detail in a later paragraph.

**Figure 1:**
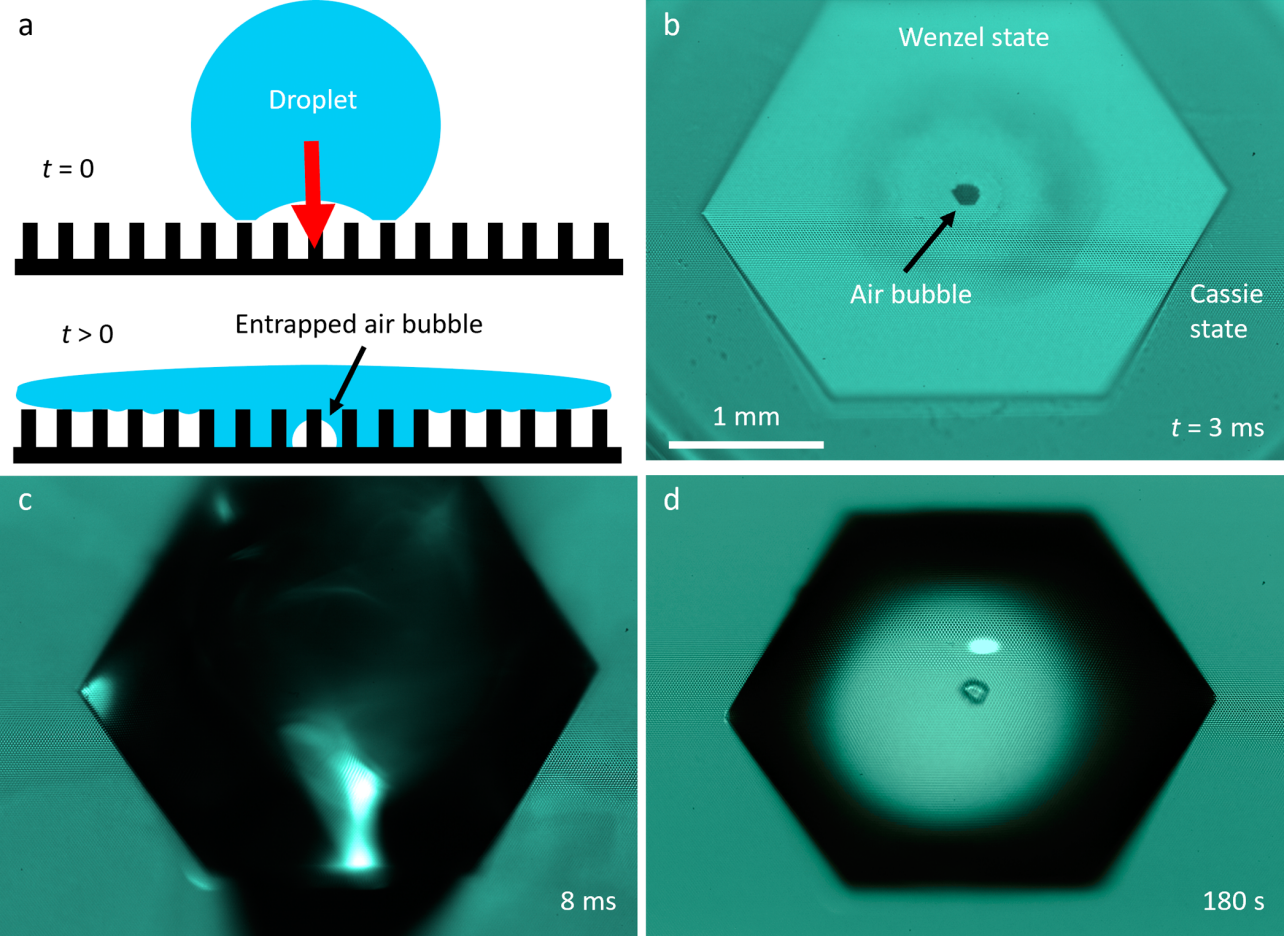
Liquid patterning using droplet impact. (a) Depending on the impact velocity, a droplet impacting a nonwetting pillar array structure can penetrate the pillars and form a fully wetted area. An air bubble is entrapped at the center of the droplet due to a stagnation point on a deformable interface. (b) The air bubble, fully wetted area (Wenzel state), and the liquid sheet gliding over the pillar top (Cassie state) can be identified by analyzing the high-speed images. (c) The fully wetted polygonal area is maintained after the radially spreading liquid sheet retracts. (d) The polygonal area is pinned and maintained as the droplet evaporates.

In a typical experiment, the entrapped air bubble at the center of the droplet is visible as shown in Figure 1b. Also visible in the top-down image of an impacting droplet are the wetted area (Wenzel state) and the area that is not fully wetted (Cassie state) where the radial spreading liquid sheet glides over the pillar tops. The radially spreading liquid lamella at time *t* = 3 ms in Figure 1b retracts back fully at time *t* = 8 ms, as shown in Figure 1c. Notably, the three-phase contact line pinning is visible during spreading (*t* = 3 ms), after the droplet has retracted (*t* = 8 ms), and after the top layer evaporated (*t* = 180 s, Figure 1d).

The shape of the wetted area is determined by the density (pillar diameter-to-spacing ratio) and arrangement (inline versus staggered) of the pillars. In the following few paragraphs, we will show that the various polygonal wetted shapes shown in Figure 2 (that is, square, octagon, rectangle, hexagon, dodecagon, and extended hexagon) can be obtained by varying the pillar dimensions (diameter and spacing) and arrangement. Importantly, we show that such shapes can be obtained by manipulating the surface tension and hence the intrinsic Young contact angle. For example, using an inline array of micropillars, a square or an octagon shape can be obtained either by varying the pillar density or by varying the Young contact angle. In a similar fashion, the pillar density and/or the Young contact angle or a combination thereof can be manipulated to obtain a hexagon or a dodecagon on a staggered array of micropillars. Rectangles and extended hexagons can also be obtained by creating an unequal spacing along the vertical and horizontal directions, that is, by varying center-to-center spacing between neighboring silicon micropillars in the horizontal and vertical directions.

**Figure 2:**
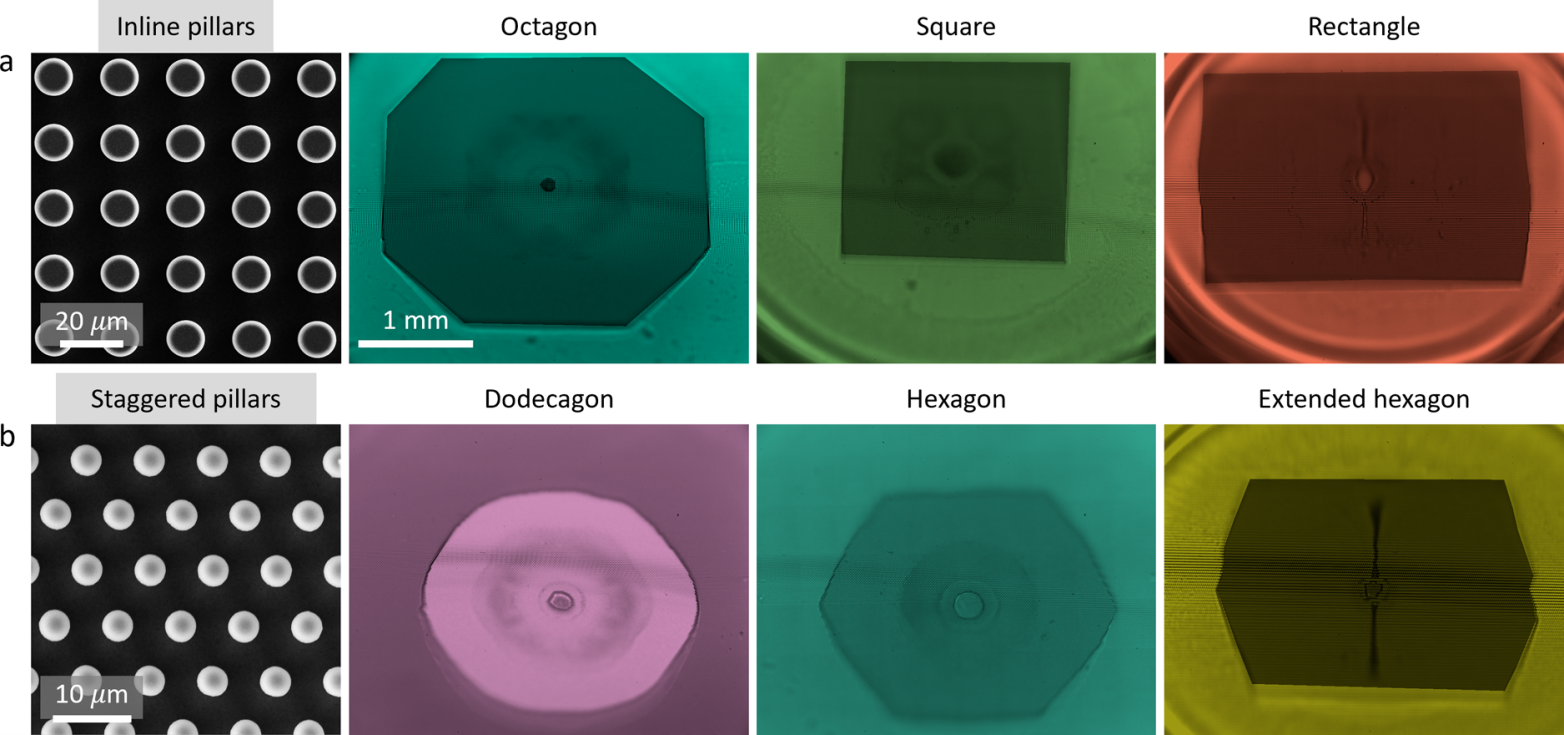
Various shapes on inline and staggered pillars. (a) The liquid–solid contact shape and wetted area are determined by the density and arrangement of the pillars (inline versus staggered). Notably, an air bubble is entrapped at the center of the wetted area. An octagon or a square is obtained using an inline arrangement of the pillars, depending on the pillar density and the advancing contact angle. (b) Similarly, a dodecagon or a hexagon is obtained on a staggered array of pillars. Rectangles and extended hexagons are obtained by varying the micropillar spacing in the vertical and horizontal directions. Outside of the wetted area, the liquid sheet radially expands in a Cassie state and retracts back, leaving behind only the fully wetted polygonal area.

To explain the difference in the wetted area shape, it is necessary to understand the motion of the three-phase contact line.^36,38^ We studied the motion of the contact line by acquiring time-lapse images at high magnification that captured the pillar-to-pillar propagation of the contact line as shown in Figure 3a. The high-resolution images were obtained using an inline array of micropillars (diameter *D* = 25 μm and center-to-center spacing *L* = 40 μm). The advancing contact angle (θ_*A*_) on the corresponding flat surface coated with C_4_F_8_ is 109°. The contact line of the wetted area (Wenzel state) is locally pinned at the pillar edges, which presents a significant energy barrier for the advancing liquid front.^69,70^ The motion of the advancing liquid front stops momentarily when it reaches the pillar top edges, as shown in the image on the first row (*t* = 2.30 ms) in Figure 3a. The contact line then depins from the pillar top at one point and jumps to the next row of pillars (*t* = 2.36 ms). Typically, such a depinning event precedes the lateral propagation and zipping motion of the contact line in the lateral direction, as shown by the arrows at time *t* = 2.45 ms and *t* = 2.51 ms in Figure 3a. The lateral zipping motion is complete at *t* = 2.57 ms, which initiates another cycle of depinning-zipping motion of the contact line for the next row of pillars. This stepwise propagation and contact line zipping is similar to the previously reported imbibition of a liquid front on a textured wetting surface.^36,71^ A highly magnified high-speed video of the zipping motion is provided in Supplementary Movie 1.

**Figure 3:**
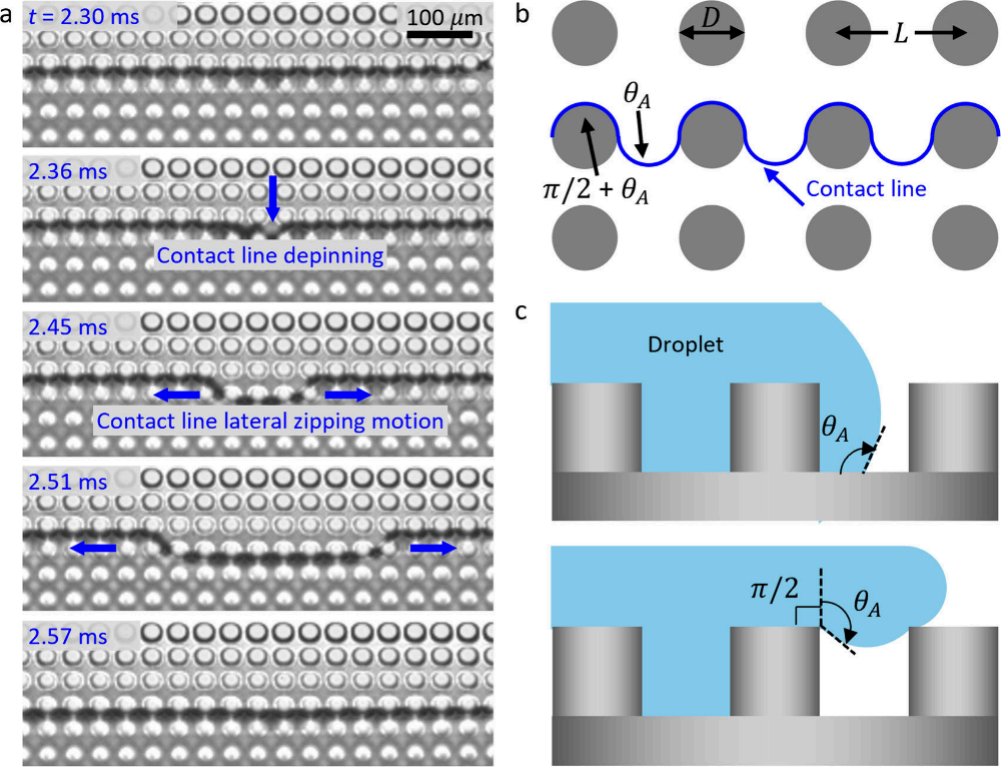
Contact line dynamics. (a) Time-lapse images of the contact line motion. The liquid front moves from the top to the bottom of the micropillars. The contact line is nearly horizontal at *t* = 2.30 ms after impact. The contact line depins from the pillar top near the center as shown by the vertical arrow at *t* = 2.36 ms, leading to zipping motion in the lateral direction in subsequent images. The left–right lateral zipping motion of the contact line that is shown by the horizontal arrows is complete at *t* = 2.57 ms, which will initiate another cycle of depinning-zipping motion of the three-phase contact line in the next row of pillars. Our high-speed videos, which are acquired at 33,000 fps, captured the depinning-zipping motion of the contact line. (b) Top-down view of the cylindrical pillar array structure. The pillar diameter and center-to-center spacing are denoted using D and L, respectively. The contact line requires bending π/2 radians beyond the advancing contact angle to reach the neighboring pillar due to the change of plane from horizontal to vertical. (c) The liquid front advances on the flat section of the pillar top and pillar base with the intrinsic advancing contact angle of the droplet on a smooth surface, θ_*A*_. Notably, the advancing contact angle at the top edge of the pillar has a barrier to overcome (horizontal-to-vertical change of plane) leading to a higher advancing contact angle, θ_*A*_ + π/2.

### Contact Line Dynamics

The apparent contact angle θ_*app*_ of a droplet on a composite/heterogeneous surface is given by the classical Cassie model^21,22,25^ using

1 $$\cos\theta_{\mathrm{app}}=f_{1}\cos\theta_{1}+f_{2}\cos\theta_{2}$$

where *f*_1_ and *f*_2_ are the area fractions of the two surfaces and θ_1_ and θ_2_ are the corresponding intrinsic contact angles of the droplet on the two background surfaces. The advancing front on a surface decorated with pillars is pinned and distorted on the pillar tops, resulting in a local advancing contact angle on the pillar tops different from that on the flat section between the pillars, as depicted schematically in Figure 3b. Accounting for the contact line pinning at the pillar edges, it was previously shown^38^ that the local advancing contact angle on pillar tops (θ_1_ in eq 1) becomes θ_*A*_ + π/2, where θ_*A*_ is the advancing contact angle on a flat surface as shown in Figure 3b and Figure 3c. This is because as the plane changes from horizontal to vertical, the contact line is required to bend π/2 radians beyond the advancing contact angle to overcome the energy barrier. Accordingly, for a Wenzel-type droplet on a surface decorated with cylindrical micropillars, the apparent advancing contact angle of the liquid front in the axial direction (θ_*A,axial*_) is modeled using,^38^

2a $$\cos\theta_{A,\mathrm{axial}}=\left(\frac{D}{L}\right)\cos\left(\theta_{A}+\pi /2\right)+\left(1-\frac{D}{L}\right)\cos\theta_{A}$$

where the *D*/*L* ratio is the linear density of the micropillars. In the diagonal direction, this equation is modified to reflect the change in pillar spacing from *L* to $\sqrt{2}L,$ and the apparent advancing contact angle (θ_*A,diag*_) becomes,

2b $$\cos\theta_{A,\mathrm{diag}}=\left(\frac{D}{\sqrt{2}L}\right)\cos\left(\theta_{A}+\pi /2\right)+\left(1-\frac{D}{\sqrt{2}L}\right)\cos\theta_{A}$$

This model captures the spreading dynamics of the three-phase contact line in the axial and diagonal directions that will lead to the different polygonal shapes reported in this study.

The observation that the liquid front advances at different contact angles in the axial and diagonal directions suggests unequal contact radius along the two directions (*a*_*axial*_ ≠ *a*_*diag*_) as shown in Figure 4a and Figure 4b. Assuming the same droplet height (*h*_1_ ≈ *h*_2_), a reasonable assumption for a spherical droplet, the ratio of the contact radii can be calculated as follows. The contact radius *a* (Figure 4c) *a* = *R* sin θ_*A*_ = *h* sin θ_*A*_/(1 – cos θ_*A*_) where *R* is the droplet radius given by R = *h*/(1 – cos θ_*A*_). The ratio of the contact radii in the axial (*a*_*axial*_) and in the diagonal direction (*a*_*diag*_) is given by

3 $$\frac{a_{\mathrm{axial}}}{a_{\mathrm{diag}}}=\frac{\sin\theta_{A,\mathrm{axial}}\left(1-\cos\theta_{A,\mathrm{diag}}\right)}{\sin\theta_{A,\mathrm{diag}}\left(1-\cos\theta_{A,\mathrm{axial}}\right)}$$

**Figure 4:**
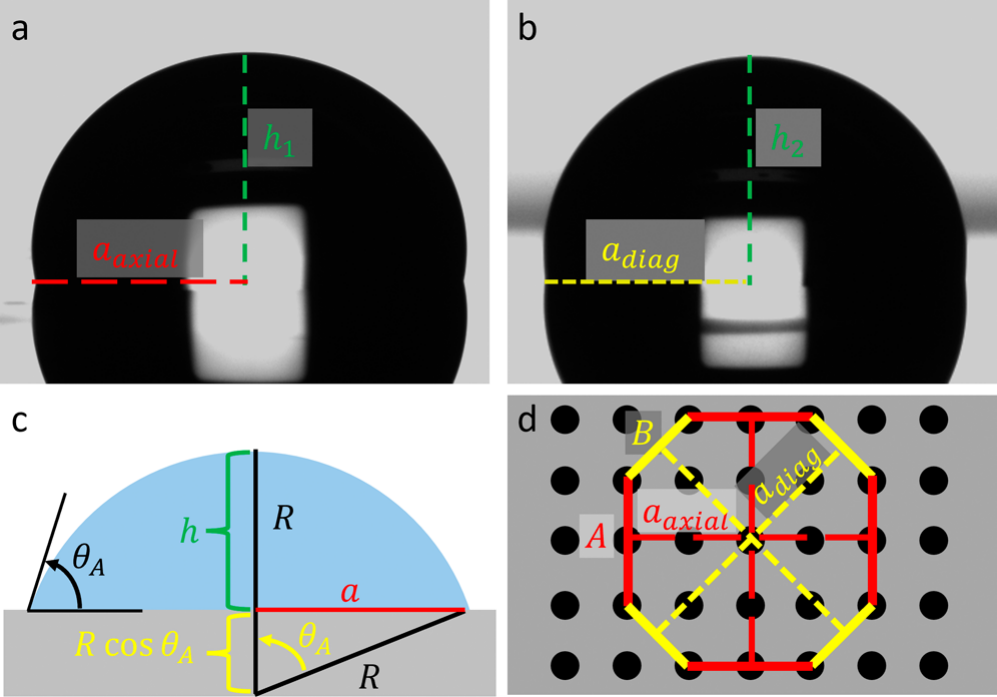
The base radii in axial and diagonal directions. (a) The base radius in the axial direction. (b) The base radius in the diagonal direction. (c) The relationship between the droplet radius *R*, contact radius *a* and droplet height *h* is shown schematically. (d) The contact radii along the lines of symmetry (axial and diagonal) are used to estimate the side ratio *B*/*A* of the resulting polygonal pattern.

The contact radii *a*_*axial*_ and *a*_*diag*_ can be written in terms of the side lengths of the sides of the polygon along the axial and diagonal direction *A* and *B* shown in Figure 4d as $2a_{\mathrm{axial}}=A+\surd 2B$ and $2a_{\mathrm{diag}}=B+\surd 2A.$ Rearranging eq 3 and using eq 2a and eq 2b for advancing contact angle along the axial direction (θ_*A,axial*_) and diagonal direction (θ_*A,diag*_), a geometry-based model to predict the side ratio emerges after rearranging the terms as^38^

4 $$\frac{B}{A}=\frac{\sqrt{2}\sin\theta_{A,\mathrm{axial}}\left(1-\cos\theta_{A,\mathrm{diag}}\right)-\sin\theta_{A,\mathrm{diag}}\left(1-\cos\theta_{A,\mathrm{axial}}\right)}{\sqrt{2}\sin\theta_{A,\mathrm{diag}}\left(1-\cos\theta_{A,\mathrm{axial}}\right)-\sin\theta_{A,\mathrm{axial}}\left(1-\cos\theta_{A,\mathrm{diag}}\right)}$$

where *B*/*A* is the side ratio shown in Figure 4d.

The contact line model shows that the shape of the wetted area is determined by the intrinsic advancing contact angle (θ_*A*_) and the linear pillar density (*D*/*L*). Note that the pillar density *D*/*L* is embedded in eq 4 since it determines the apparent advancing contact angles in the axial and diagonal directions (θ_*A,axial*_ and θ_*A,diag*_) as given by eq 2a and eq 2b. Based on our contact line model shown in eq 4, a square shape of the wetted area (*B*/*A* ≈ 0) is expected when θ_*A*_ is low (θ_*A*_ ≈ 85°), whereas a regular octagon shape (*B*/*A* ≈ 1) is expected when θ_*A*_ is high (θ_*A*_ ≈ 125°). The relationship between the *B*/*A* ratio and the advancing contact angle θ_*A*_ is shown in Figure 5a. We selected two *D*/*L* ratios to show the theoretical trend: a blue solid line for sparse pillars with *D*/*L* = 0.53 and a red dotted line for dense pillars with *D*/*L* = 0.67. For the experiment, we varied the advancing contact angle between 80° and 125° by adding ethanol to deionized water. The blue crosses, which are obtained experimentally, correspond to the blue solid theoretical line for *D*/*L* = 0.53. Similarly, the experimentally obtained red triangles correspond to the theoretical red dotted line for *D*/*L* = 0.67. The reasonably good agreement between the theoretical prediction and the experimental data points shows that our contact line model captures the contact line motion and the resulting shape of the wetted area during droplet impact. As shown by the inset in Figure 5a, the shape of the wetted area is a square (*B*/*A* ≈ 0) when the advancing contact angle is low (θ_*A*_ ≈ 95°) whereas the shape of the wetted area becomes an octagon (*B*/*A* ≈ 1) when the advancing contact angle is high (θ_*A*_ ≈ 125°).

**Figure 5:**
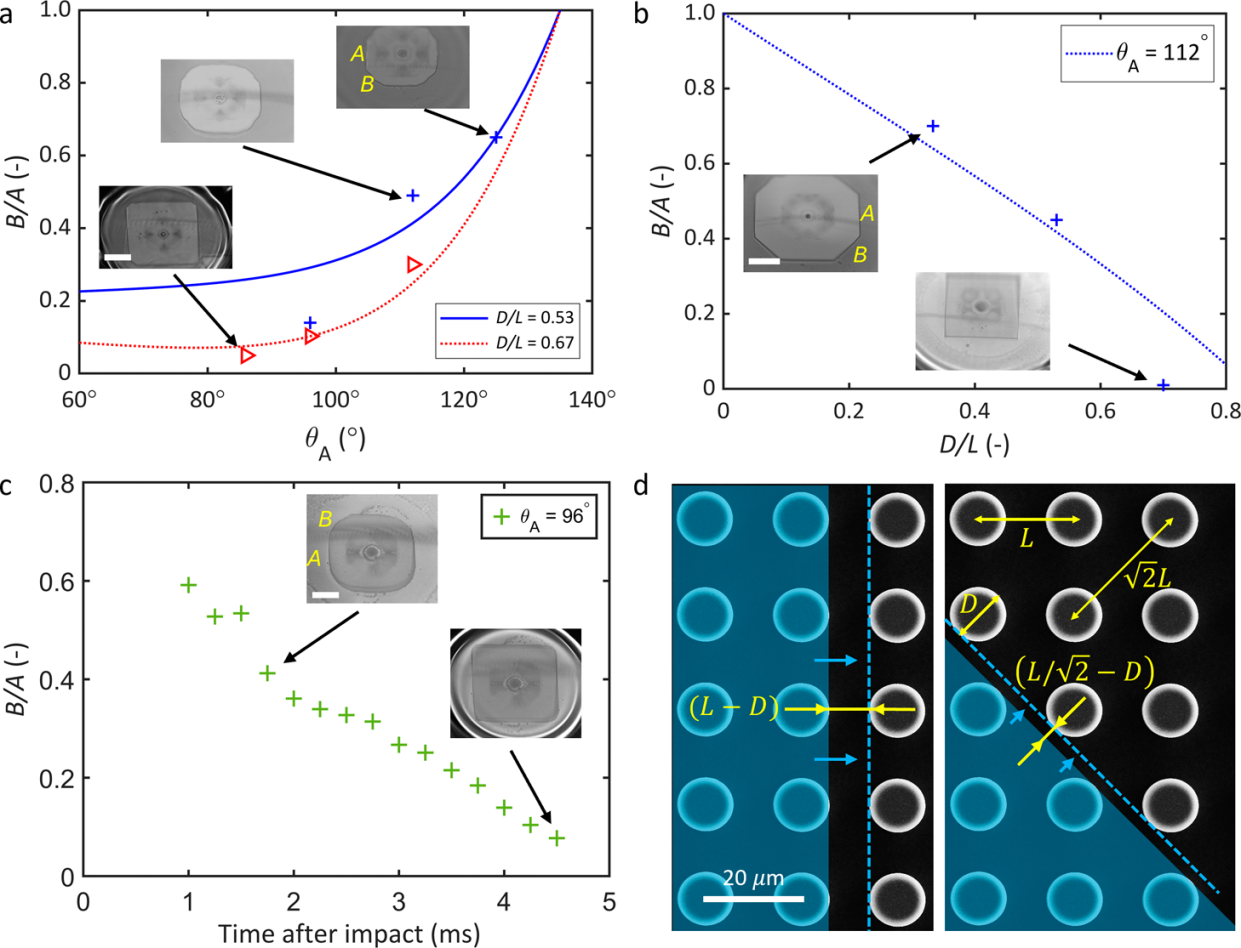
Evolution of the contact area. (a) Comparison of experimental results and model prediction of the ratio of the diagonal to the axial sides of an octagon (*B*/*A*) on a square array with varying advancing contact angle. The experimental results, which agree well with our analytical model, show that *B*/*A* increases with the advancing contact angle of the droplet on the smooth counterpart. (b) Pillar density also affects the shape of the resulting polygon during impact. As the *D*/*L* ratio increases, the polygon becomes closer to a square. (c) The ratio B/A changes with time on dense pillar arrays due to the difference in the propagation speed of the liquid front (or contact line) along the axial and diagonal directions. (d) The interpillar spacings for the liquid front moving in the axial and diagonal directions are (*L* – *D*) and $\left(L/\sqrt{2}\right)-D,$ respectively. The interpillar distance and solid area along the axial direction are larger than diagonal direction, which results in a faster motion of the contact line along the diagonal direction. Scale bar, 1 mm.

The theoretical prediction of the effect of the linear pillar density *D*/*L* on the side ratio *B*/*A* for an advancing contact angle θ_*A*_ = 112° is shown by the dashed blue line in Figure 5b. In our experiments, we varied the *D*/*L* ratio from 0.3 to 0.7 by systematically varying the pillar diameter and spacing during the sample fabrication stage that involves contact photolithography and deep reaction ion-etching. The experimental data points in Figure 5b show good agreement with our closed-form analytical solution of the contact line dynamics. In our experiments, the shape of the wetted area was an octagon when the pillar density was low (*D*/*L* ≈ 0.33) whereas it became a square for high pillar density (*D*/*L* = 0.67). We attribute the slight deviation of the experimental data point for *D*/*L* = 0.67 from the theoretical prediction in Figure 5b to the variation in the speed of propagation of the three-phase contact line in the axial and diagonal directions.

To better explain the slight mismatch between experiment and contact line mode, we monitored the evolving shape of the wetted area when a millimeter-sized water droplet impinges on a surface that has θ_*A*_ = 96°. The resulting contact line motion and the resulting wetting area shape as a function of time are shown in Figure 5c. For this analysis, we captured high-speed images at 10,000 frames-per-second (fps). The side ratio *B*/*A* changes with time as the three-phase contact line, and hence the solid–liquid contact shape, continues to spread in the axial and diagonal directions at different speeds. Based on the current understanding of the pinning-depinning motion of the contact line, the speed at which the radially expanding liquid front reaches the next row of pillars determines the speed of the liquid front. The inner-pillar distance (edge-to-edge pillar spacing shown schematically in Figure 5d) is shorter along the diagonal direction $\left(L/\sqrt{2}-D\right)$ than along the axial direction (*L* - *D*). This variation leads to faster pillar-to-pillar propagation of the contact line in the diagonal direction (shortest path) than the axial direction (longest path). Note that the edge-to-edge spacing between neighboring pillars along the diagonal direction $D/\sqrt{2}L$ is smaller than the edge-to-edge pillar spacing along the axial direction *D*/*L*. This geometry and arrangement of pillars favors the motion of the contact line, and hence the radially expanding liquid front, along the diagonal direction than the axial direction.

In addition to the shape, the solid–liquid contact/pattern size of an impinging droplet on a surface has important implications, for example, in jet impingement cooling in two-phase heat transfer. Our experiments show that the pillar geometry and droplet viscosity affect the size of the contact area. The contact area decreases as the pillar density increases, as shown by the two insets in Figure 5b where the square formed on dense pillars (*D*/*L* = 0.7) is smaller than the octagon formed on sparse pillars (*D*/*L* = 0.3). Our experiments also show that the size of the contact area decreases as the droplet viscosity increases due to the increased viscous dissipation as the liquid front propagates through the pillar array porous structure. Increasing the droplet viscosity has a similar effect to increasing the pillar density on the size of the contact area. Increasing the pillar density increases viscous dissipation by decreasing the size of the pores available for liquid transport. Furthermore, our experiments show that the size of the contact area increases with the Weber number. Detailed description of the effect of droplet viscosity and Weber number on the spreading dynamics is provided in Supporting Information S4.

### Entrapped Air Bubble

We now turn our focus to the air bubble entrapped underneath the liquid sheet and consider the evolution of the bubble on the textured surface with varying pillar density and arrangement. The entrapped air bubbles on an inline array of various pillar densities are shown in Figure 6a–c. When the pillar density is low (*D*/*L* = 0.33), the relatively large spacing between the pillars allows the air film to retract to the center to form one large air pocket. As the pillar density increases, however, the resistance to the retraction of the entrapped air film increases, resulting in bubble fragments forming microbubbles pinned to the pillar tops. In our experiments, when the pillar density *D*/*L* = 0.67, most of the air film retracts to form a central bubble while the rest of the film forms a ring of microbubbles around the large bubble, as shown in Figure 6b. On high-density pillars (*D*/*L* = 0.83), the air film is entirely fragmented into smaller microbubbles, as shown by the arrow in Figure 6c. Such dependence of the air bubble size on the pillar density can be rationalized by considering how the air film beneath a falling droplet forms an air bubble. It was shown previously that an air film of thickness ≈1 μm is entrapped underneath the droplet, which retracts to the center of impact to minimize surface energy.^42,72,73^ When the pillar density is low, the air film is able to retract to the center through the sparse pillars to form one large bubble. On dense pillars, however, the increased resistance prohibits full retraction of the air film, resulting in fragmented microbubbles shown by the arrow in Figure 6c. The appearance of multiple microbubbles on pillar tops has been previously reported.^40^

**Figure 6:**
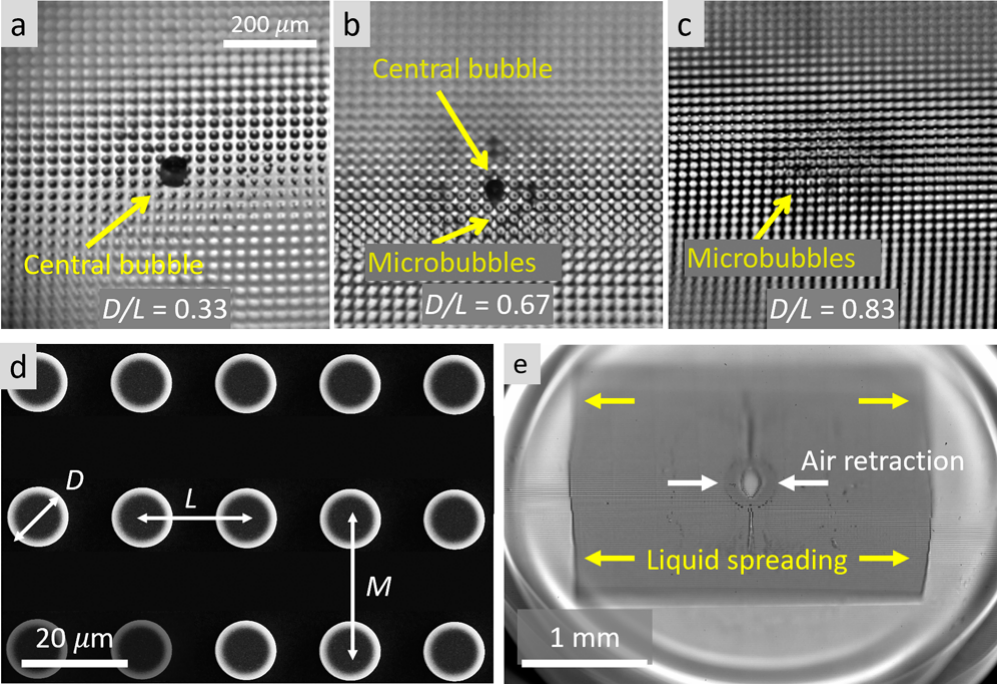
Entrapped air shape and retraction mechanism. (a) For sparse pillars (*D*/*L* = 0.33), the entrapped air film retracts toward the center of the droplet to form one large air bubble shown by the arrow. (b) For *D*/*L* = 0.67, most of the air film retracts to form one central bubble, while some of it fragments to form microbubbles around the central bubble. (c) For dense pillars (*D*/*L* = 0.83), the entrapped air film fails to retract fully to the center, hence a lack of central bubble. However, the fragments are still visible, forming a larger ring that is formed by fragments of microbubbles on pillar tops, as shown by the arrow. (d) An SEM image of a cylindrical pillar array with diameter *D*, horizontal spacing *L*, and vertical spacing *M*. The dimensions for *D*, *L*, and *M* are varied systematically to capture distinct wetting patterns and bubble retraction mechanisms. (e) The liquid pattern formed by the wetted area is elongated in the horizontal direction when *M* > *L*. The opposite effect is observed on the entrapped bubble, showing the universality of the impact of pillar density on contact line dynamics.

The dependence of the liquid pattern and air bubble on pillar spacing suggests elongated liquid patterns and air bubbles on pillar arrays with different vertical and horizontal spacings. We investigated this on rectangular pillar arrays with diameter *D* = 8 μm, horizontal spacing *L* = 10 μm, and vertical spacing M = 15 μm as shown in Figure 6d. Notably, the anisotropic spacing in rectangular pillar arrays has an opposing effect on the entrapped air bubble and wetted solid–liquid contact area. The air bubble, which is formed due to retraction of an air film toward the center, has a smaller horizontal dimension than the vertical dimension when *M* > *L*. On the other hand, the liquid front, which spreads outward, has a larger horizontal side than vertical side, again affected by the pillar arrangement. This opposite yet complementary result highlights the universality of our interpretation of eq 4 for both the liquid pattern and bubbles.

Finally, we compared the shapes of contact areas obtained by high-speed droplet impact on nonwetting surfaces with the shapes obtained by gentle deposition of Wenzel-type nonimbibing droplets. This comparison is shown in Figure 7. The wetted area obtained during impact is pinned at the base of the pillars, and the shape is maintained even after full retraction and eventual evaporation of the spreading liquid sheet, as shown in Figure 7a. The liquid shapes obtained via impact are similar to those obtained via evaporation of sessile droplets on wetting surfaces (Wenzel-type droplets) shown in Figure 7b. The only noteworthy difference in the two scenarios is the presence of an entrapped air bubble under the impacting droplet, which is absent when the droplet is deposited gently on the wetting surface. The similarity of the patterns obtained via impact on nonwetting surfaces and via deposition on wetting surfaces is crucial for various applications, as it enables the printing of liquid patterns on nonwetting surfaces. Previously, the formation of faceted droplets on superhydrophobic surfaces has been shown,^59^ but only temporarily since the droplets retracted to eventually bounce off the surface.

**Figure 7:**
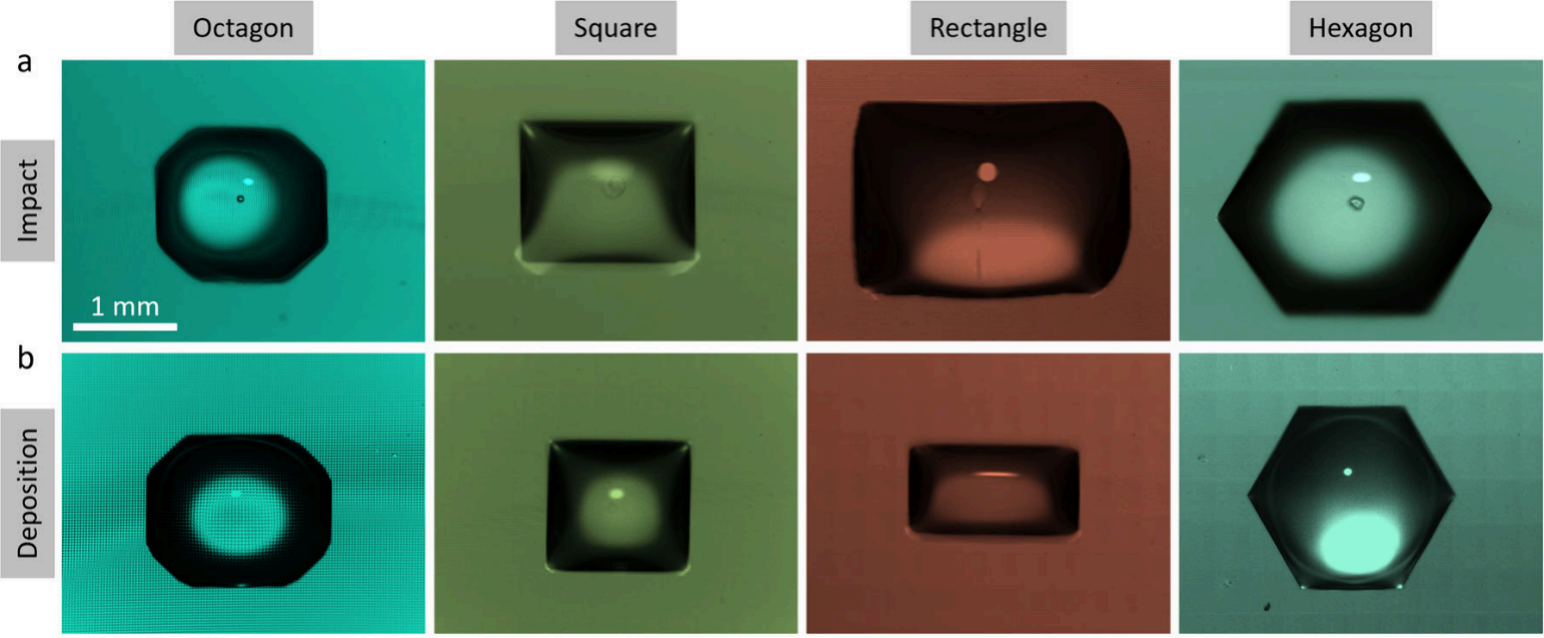
Similarity of liquid patterns. The liquid patterns obtained using (a) high-speed droplet impact on textured nonwetting surfaces and (b) evaporation of nonimbibing Wenzel-type sessile droplets on wetting surfaces are similar, with one major difference. Unlike the slow droplet deposition, the high-velocity droplet impact entraps an air bubble at its center due to the maximum local pressure, which deforms the center of the droplet at its base.

## Conclusion

In summary, we demonstrate the ability to control and manipulate the shape of the Wenzel-type wetted area when a droplet impacts a micro/nanotextured nonwetting surface. This unique approach enables the deposition of distinct polygonal liquid patterns on well-defined silicon micropillars. Our experiments show that the shape of the wetted area during droplet impact is influenced by the pillar density (diameter-to-spacing ratio), pillar arrangement (inline versus staggered), and/or the advancing contact angle of the droplet. By varying the pillar density and/or the advancing contact angle (by virtue of changing the surface tension of deionized water by adding ethanol), we show that square and octagon Wenzel-type wetted area shapes form on inline pillars while the wetted area morphology changes to hexagon and dodecagon on staggered pillars. Rooted in the fundamentals of contact line physics, our closed-form analytical model shows accurate prediction of the shape of the wetted area for given impact dynamics and micropillar array arrangement. Furthermore, this study shows that the same dynamics that controls the wetted area morphology dictates the shape of the entrapped air bubble underneath a droplet during high-velocity droplet impact. A central bubble forms on sparse pillars while fragmented microbubbles form on dense pillars. The shape of the wetted area resulting from high-velocity droplet impact is identical to the liquid pattern that an evaporating sessile droplet forms. This work is distinct from previous studies in four major areas. First, our surfaces are textured nonwetting or superhydrophobic while prior works used textured wetting surfaces. Second, our work uses high-velocity droplet impact to penetrate the micropillars and pattern the Wenzel-type wetted area while past studies employed evaporation of sessile droplets as the main strategy to patten the solid–liquid contact area/shape. Third, in this study we used pillar density and advancing contact angle as parameters to manipulate the three-phase contact line dynamics to achieve the desired wetting morphology. Past studies, on the other hand, used only pillar density for liquid patterning of an evaporating sessile droplet. Therefore, this is the first work that reports the effect of advancing contact angle on liquid patterning. Fourth, unlike past studies which only investigated steady-state shape, this work reports both the steady-state and the transient wetting morphology of the solid–liquid contact area of an impacting droplet. This robust and accurate strategy to form high-resolution liquid patterns on nonwetting surfaces demonstrates a unique approach for facile and controllable deposition of liquids on solid surfaces. The unique approach reported in this work has promising applications in liquid-based printing technologies such as inkjet printing of polymer dots and biomicroarrays and spray cooling for electronics thermal management.

## Methods

### Sample Fabrication

Standard contact photolithography and deep reactive ion etching (DRIE) were used to fabricate the well-defined silicon micropillars used in this study. Following DRIE, the micropillars were coated with a ≈100 nm thick hydrophobic layer of octafluorocyclobutane (C_4_F_8_) to make them water repellent (intrinsic contact angle = 113° ± 2°). Before the start of each experiment, the substrates were rinsed with solvents (acetone, methanol, IPA, and deionized water). The scanning electron microscope (SEM) images of the micropillars are shown in Supporting Information S1.

### Contact Angle Measurements

The static and advancing contact angle of water droplets on the fabricated samples was measured using a drop shape analyzer (DSA100 Expert, Kruss GmbH). The static contact angle was measured by analyzing the shape of a sessile water droplet (≈10 μL) on the textured surface. The advancing contact angle was measured by adding more water to the sessile droplet at a slow infusion rate (0.1 μL/s). More details of the contact angle measurement strategy and protocol are presented in Supporting Information S3.

## Supplementary Material





## Notes

Details of sample fabrication, experimental setup, contact angle measurement, and effects of droplet viscosity and Weber number (PDF)
Highly magnified view of the propagation of the fully wetted liquid front at the pillar scale (MP4)
