# Supplementary material for: Liquid Patterning Using Droplet Impact on Textured Nonwetting Surfaces
Source: ACS Appl Mater Interfaces. 2026 Jan 20;18(5):9206–15. doi: 10.1021/acsami.5c23079 (PMC12903111; doi:10.1021/acsami.5c23079)
Supplement: Supplementary file 1 [file am5c23079_si_001.pdf]

Supplementary Information for
Liquid patterning using droplet impact on textured non-wetting surfaces

Biruk Teka Gidreta[1], Elijah Williams[1], Michal Remer[1,2], Solomon Adera[1*]
[1]Energy Transport Lab (ETL), Department of Mechanical Engineering, University of Michigan, Ann Arbor, Michigan, USA
[2]Institute of Aeronautics and Applied Mechanics, Warsaw University of Technology, Warsaw, Poland
*Corresponding author: Solomon Adera
Email: sadera@umich.edu

Supplementary movies

Movie 1. Contact line dynamics. This movie shows the propagation of the fully wetted liquid front at the pillar scale. The contact line propagates from one row of pillars to the next in a depinning-zipping motion. The movie is played by slowing the impact process by 3,300×

S1. Sample fabrication

In this study, we first fabricated silicon microstructures using standard contact photolithography and deep reactive ion etching. The microstructures are well-defined cylindrical micropillars arranged in an inline isotropic (Figure S1a), inline anisotropic (Figure S1b), and staggered (Figure S1c) pattern. The pillar arrays were then coated with a 150 nm thick layer of octafluorocyclobutane (C_4F_8) to increase their liquid repellency. The micro-pillars have diameters D = 5–15 μm, center-to-center spacing L = 8–20 μm, and height H = 10 μm. The scanning electron micrograph images (Mira3 FEG-SEM, Tescan) of the pillar array structure are shown in Figure S1. The top panels show the top-view SEM images while the bottom panels show images captured at 45° tilt angle.

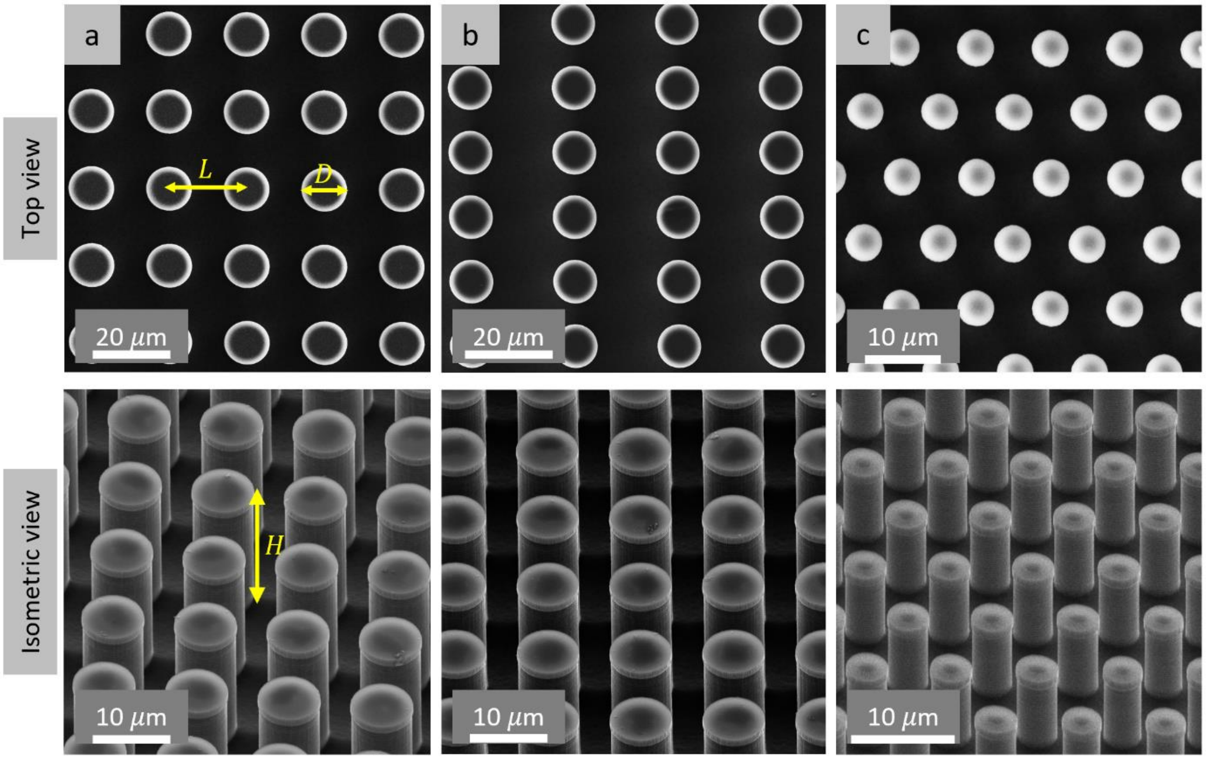

Figure S1. Scanning electron micrograph (SEM) images. SEM images of silicon micropillars in (a) inline isotropic, (b) inline anisotropic, and (c) staggered arrangement. The top panel is the top-down view, while the bottom panel is the isometric view captured at 45° tilt angle. The micropillars are coated with octafluorocyclobutane (C_4F_8) to increase their liquid repellency. Depending on the spacing, in-line arrangement of the micropillars can lead to a square or octagon wetted area while staggered arrangement of the pillars can lead to hexagons and dodecagons.

S2. Experimental setup
In this study, high-speed images were captured at 5,000 - 33,000 frames-per-second (fps) at 1200×800 resolution to capture the droplet impact dynamics with high temporal resolution. The high-speed camera (Phantom v1610, Vision Research) is equipped with a 10× infinity corrected long working distance objective lens (Mitutoyo) to increase spatial resolution. The camera is positioned at 65° from the horizontal direction to capture the top-view of the droplet impact. A high-intensity MI-150 fiber optic light source (DOLAN JENNER Fiber-Lite) was used to illuminate the background. In a typical experiment, a 10 µl droplet is released from a known height using an ultra-precision microinjection syringe (Ultra Micro Pump III, WPI). The release height of the droplet was varied in our experiments to obtain the desired Weber number (We). The droplet is released from the syringe into a hollow square acrylic tube. The transparent acrylic tube was needed to minimize the effect of air current, which can cause the droplet to veer off target. A schematic of the experimental setup (not to scale) is shown in Figure S2.

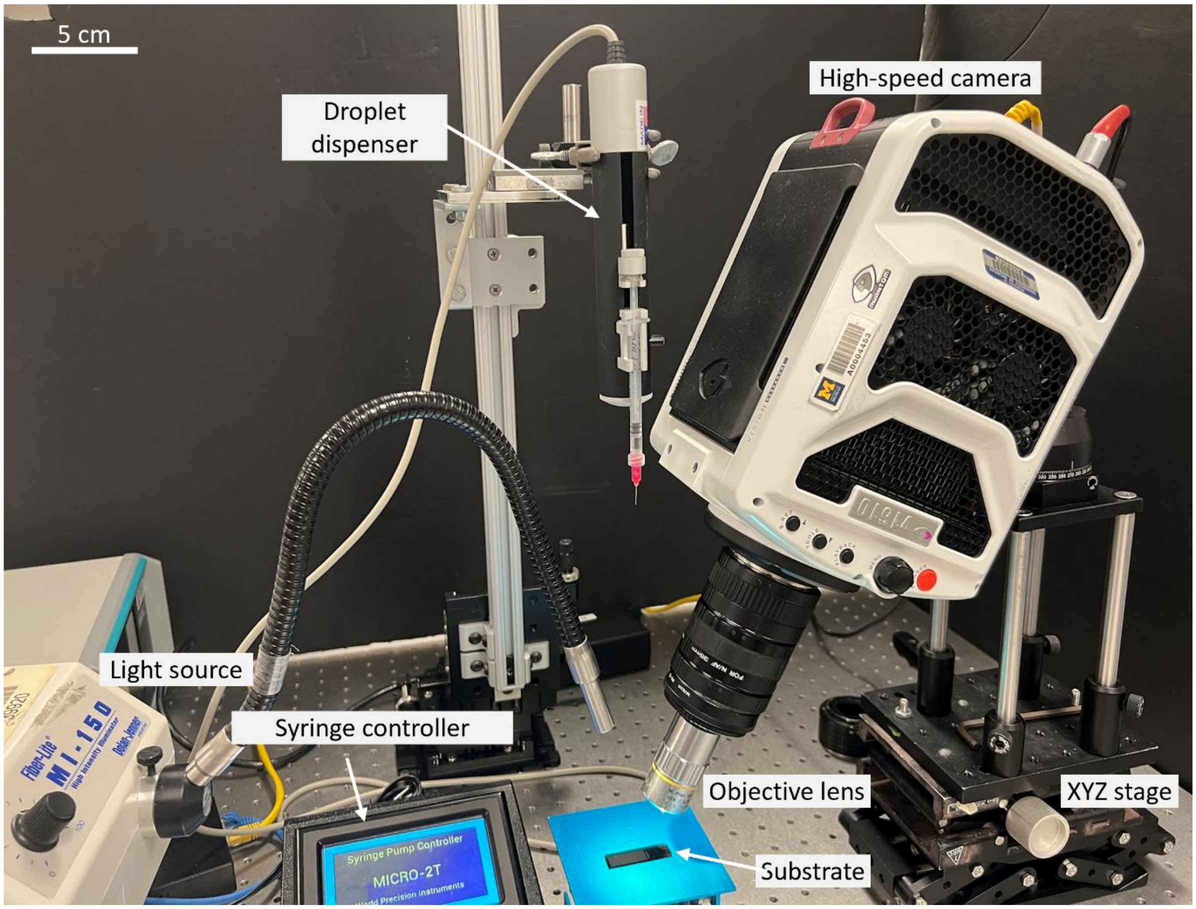

Figure S2: Experimental setup. A schematic of the experimental setup (not-to-scale) shows a high-speed camera, light source, micro-syringe, syringe controller, objective lens, and the substrate. To minimize the impact of the air draft in the laboratory, the falling droplet was contained in a transparent housing that was securely attached to a vertical stand. The high-speed camera was positioned at 65° inclination from the horizontal to capture the top view images of the impacting droplet.

S3. Contact angle measurement: The dynamic contact angle on the surfaces was measured using a drop shape analyzer (DSA100 Expert, KRÜSS GmbH). The contact angle measurements were performed at room temperature (≈23 °C) under ambient laboratory conditions. A ≈10 µl droplet was deposited on the test sample at a dispensing rate of 0.1 µl/s to minimize inertial effects during droplet formation. The side-view profile of the droplet was recorded in real time using the built-in high-speed camera of the drop shape analyzer, and the contact angle was determined by fitting the droplet shape to the Young–Laplace

equation. The Young's contact angle of water on the surfaces was 113 ± 2°. The advancing and receding contact angles were obtained by slowly increasing and decreasing the droplet volume, respectively, allowing quantification of contact angle hysteresis.[1-3]

To systematically vary the intrinsic wettability of the liquid, water–ethanol mixtures with different ethanol volume fractions were prepared. Since ethanol lowers the surface tension of the liquid, increasing the ethanol concentration led to a reduction in the apparent contact angle on the same surface. This approach allowed us to generate droplets with a wide range of equilibrium contact angles while maintaining identical surface microstructures. By tuning the intrinsic contact angle through the liquid composition, we were able to investigate how the intrinsic advancing contact angle of a droplet affects the spreading dynamics and the resulting shape of the wetted contact area during droplet impact. The advancing contact angles of the different ethanol water mixtures ranged from 85° to 125°. The contact angles of some water-ethanol mixtures are shown in Figure S3.

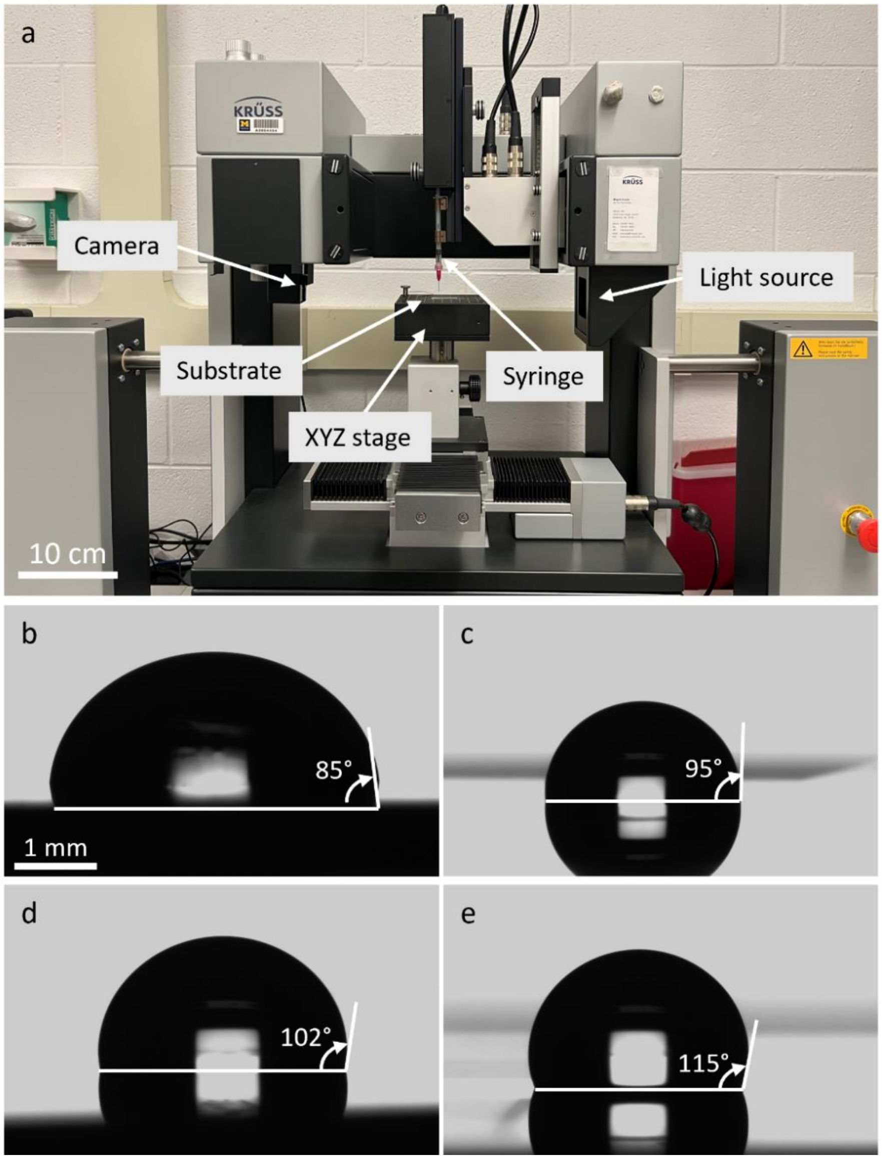

Figure S3: Contact angle measurements. (a) The drop shape analyzer used in this study. (b-e) The Young contact angle of the various water-ethanol mixtures used in this study ranges from 85° to 115°. The volume percentage of each mixture is (b) 50% ethanol– 50% deionized water, (c) 25% ethanol– 75% deionized water, (d) 10% ethanol– 90% deionized water, and (e) pure deionized water.

The static, advancing, and receding contact angles of the various water-ethanol mixtures used in this study are shown in Table S1. The measurements on the table are averages of repeated experiments with ±2° measurement uncertainty.

Table S1: Contact angle measurements. The static, advancing, and receding contact angles of the various liquids used in this study are measured using a drop shape analyzer (DSA100 Expert, KRÜSS GmbH). The liquids are obtained by mixing different volumes of ethanol and deionized water. Increasing the ethanol fraction lowers the surface tension of the liquid, which results in smaller contact angle.

Liquid	Static contact angle (°)	Advancing contact angle (°)	Receding contact angle (°)	Contact angle hysteresis (°)
Deionized water (DIW)	115	123	101	22
10% Ethanol & 90 % DIW	102	110	87	23
25% Ethanol & 75 % DIW	95	101	79	22
50% Ethanol & 50 % DIW	85	89	67	23

S4. Effect of droplet viscosity on contact area

We conducted droplet impact experiments using droplets of various viscosities (η). We used water-glycerol mixtures of various weight percentages to vary the viscosity of the droplet. Our experiments show that when the droplet viscosity increases from 1 mPa·s (Figure S4a) to 11 mPa·s (Figure S4b) and finally to 23 mPa·s (Figure S4c), the size of the fully wetted contact area decreases progressively. As discussed in the manuscript in detail, the wetted Wenzel-type contact area moves as the contact line progresses radially outwards through the pillars from one row to the next via three-phase contact line zipping motion. The speed of the contact line is governed by the complex interplay between inertia, capillary, and viscous forces.[4,5] As the viscous resistance increases with increasing viscosity, the velocity of the liquid front decreases, which results in reduction of the wetted contact pattern area. The experimental results also show that at higher viscosities, the droplet contact area remains circular. We attribute this to the increased viscous resistance experienced by the spreading liquid front. Note that increasing the viscosity of the droplet has a similar effect to increasing the density (that is, diameter-to-spacing ratio) of the silicon micropillars. Increasing the density of the pillars increases the available area for viscous dissipation, hence increased resistance to contact line motion.

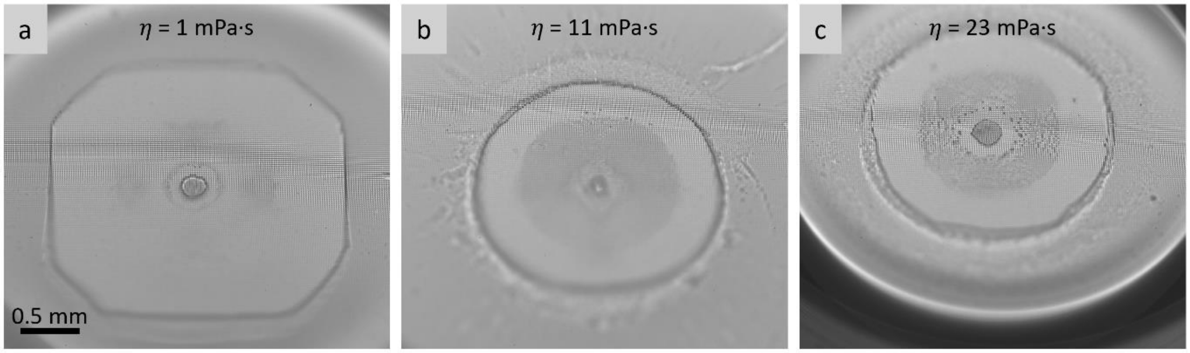

Figure S4: Effect of viscosity. As the droplet viscosity increases, the size of the fully wetted contact area decreases. (a) For a water droplet with viscosity η = 1 mPa·s, an octagonal wetting area is formed on a silicon surface that is decorated with inline silicon micropillars. (b) When the droplet viscosity increases to η = 11 mPa·s, the wetted contact area decreases. (c) Further increasing the viscosity to η = 23 mPa·s decreases the wetted area even further. As the viscosity increases, the contact pattern footprint becomes closer to a circle that we attribute to increase in viscous dissipation. Dense pillars (that is, higher diameter-to-spacing ratio) and large droplet viscosity have the same effect in terms of reducing the solid-liquid contact pattern area.

We conducted experiments by varying the height from which the droplet was released to obtain Weber numbers that range from 30 to 300. The experimental results show that the size of the fully wetted Wenzel-type contact area increases with the Weber number as shown in Figure S5. When the We < 30, the liquid patterns did not form on the micropillar array structure. Instead, we observed complete rebound of

the impacting droplet from the textured surface. We attribute this to the low inertial force of the impinging droplet not having sufficient kinetic energy to overcome the viscous resistance rendered by the side walls of the well-defined silicon micropillars. As a result, the outer surface of the droplet stretches and forms a sheet of liquid that eventually retracts and recoils from the surface due to the action of surface tension. Such droplets do not form a Wenzel-type solid-liquid contact area during impact. When the Weber number > 30, the impacting droplet penetrates the pillar array structure since the inertia of the droplet overcomes the viscous resistance rendered by the pillar side walls. In such cases, the droplet forms a Wenzel-type wetted area even though the original surface is intrinsically non-wetting.

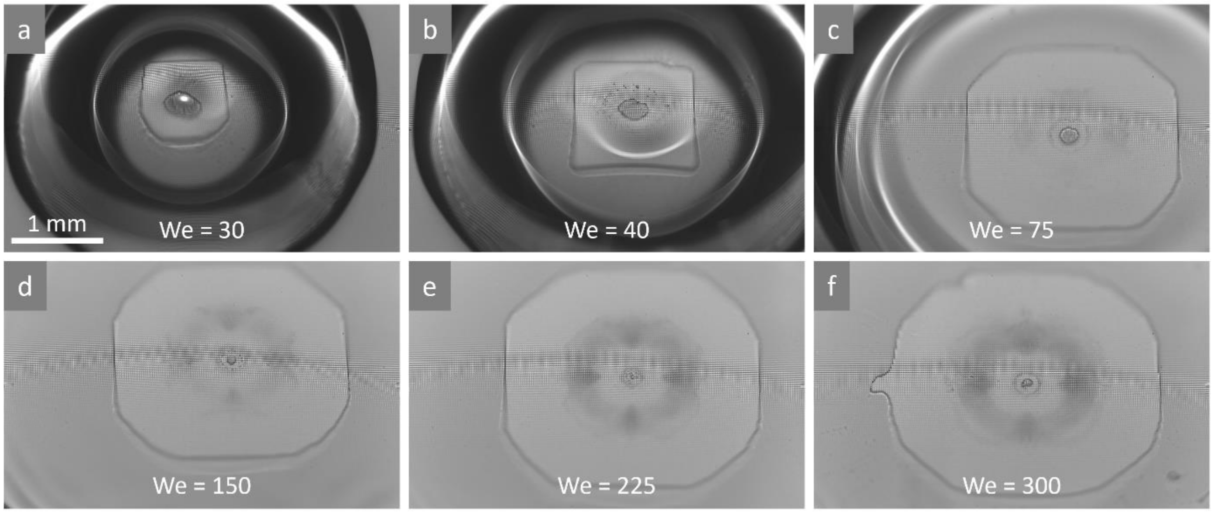

Figure S5: Effect of Weber number. A droplet impacting an inline array of pillars at different Weber number forms patterned areas of various sizes. For We < 30, no liquid pattern was formed, and the droplet completely rebounded from the surface as the kinetic energy is not sufficient to force transition to the Wenzel state. For We > 30, the droplet kinetic energy forces a transition to the Wenzel state at the center of droplet impact. This wetted contact area increases with increasing Weber number. The Wenzel-type wetted area for an impacting droplet with (a) We = 30, (b) We = 40, (c) We = 75, (d) We = 150, (e) We = 225, and (f) We = 300 increases monotonically with Weber number.

S5. Droplet contact area resolution

The liquid patterns we obtained have high resolutions that correspond to the center-to-center spacing of the pillars as shown in Figure S6. This is because the contact line moves in depinning-zipping motion from one row of pillars to the next. The liquid front needs to overcome the high energy barrier on pillar tops, as discussed in detail in the main article. This governs the motion of the contact line and hence the resolution of the liquid pattern.

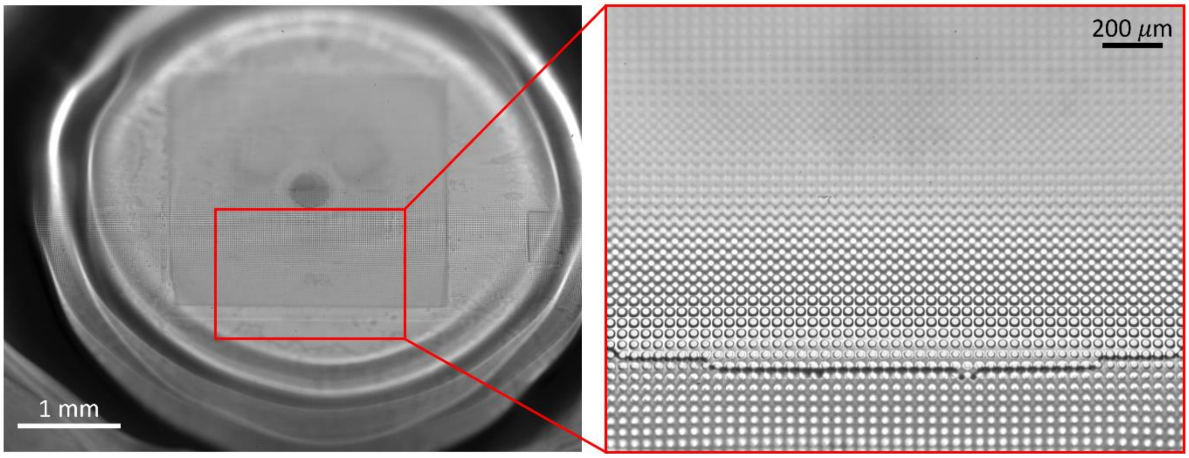

Figure S6: Droplet pattern resolution. The shape of the contact area has a high resolution that corresponds to the pillar center-to-center spacing. The wetted contact area forms after impact as the three-phase contact line propagates through the pillar porous structure. The contact line moves in a depinning-zipping motion from one row of pillars to the next. The pillar diameter, arrangement, and spacing determines the size and pattern of the polygonal solid-liquid contact shape or Wenzel-type wetted area that forms post-impact. As a result, the resolution of the liquid pattern depends on pillar size, arrangement, and spacing.

S6: Contact line dynamics for staggered pillars

The geometric argument and contact line dynamics for staggered pillars and inline pillars are identical. Similar to inline pillars, a hexagonal array has two principal axes, one principal axis parallel to the well-defined pillar array structure and a second principal axis 30° from the horizontal row of micropillars. A simple geometry analysis shows that the diagonal side has a length of $2\left(L\cos(30°)\right) = \sqrt{3}L$ when the axial center-to-center spacing between neighboring pillars is L as shown in Figure S7a. There are three axial and three diagonal axis of symmetry between 0° and 360° as shown by the schematic in Figure S7b. Accordingly, a dodecagon (12-sided polygon) forms on sparse pillars whereas a hexagon forms on dense pillars.

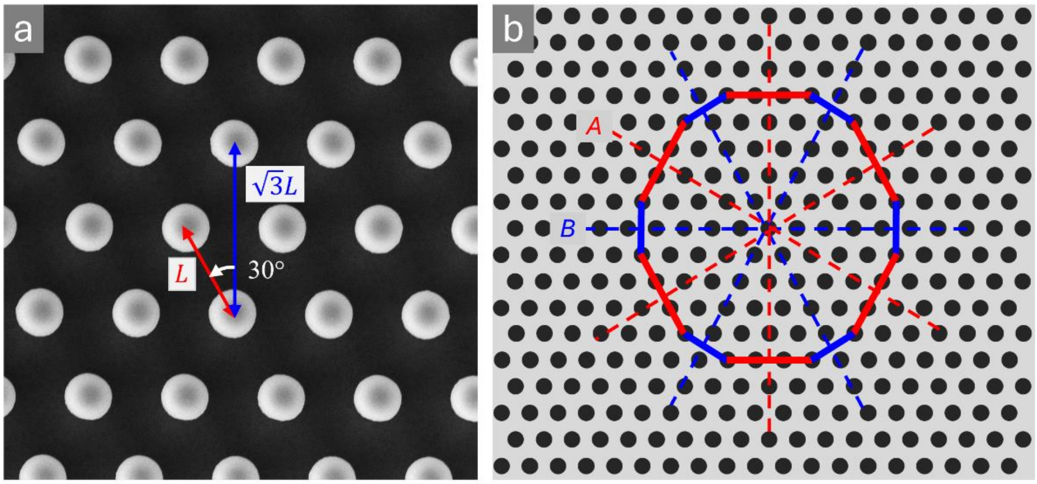

Figure S7: Contact line dynamics. The contact line dynamics and the liquid pattern are identical for staggered and inline pillars. (a) When the center-to-center micropillar spacing in the axial direction is L, the diagonal spacing becomes $2\left(L cos(30°)\right) = \sqrt{3}\mathrm{L}$. (b) There are three principal axial and three principal diagonal axes of symmetry, resulting in a dodecagon or hexagon depending on the pillar density and the contact angle of the droplet.

References

(1) Eral, H. B.; 't Mannetje, D. J. C. M.; Oh, J. M. Contact angle hysteresis: a review of fundamentals and applications. *Colloid Polymer Sci.* **2013**, *291* (2), 247-260.
(2) Gao, L. C.; McCarthy, T. J. Contact angle hysteresis explained. *Langmuir* **2006**, *22* (14), 6234-6237.
(3) Raj, R.; Enright, R.; Zhu, Y.; Adera, S.; Wang, E. Unified Model for Contact Angle Hysteresis on Heterogeneous and Superhydrophobic Surfaces. *Langmuir* **2012**, *28*, 15777-15788.
(4) Courbin, L.; Denieul, E.; Dressaire, E.; Roper, M.; Ajdari, A.; Stone, H. A. Imbibition by polygonal spreading on microdecorated surfaces. *Nat. Mater.* **2007**, *6* (9), 661-664.
(5) Su, J. P.; Legchenkova, I.; Liu, C.; Lu, C. G.; Ma, G. Y.; Bormashenko, E.; Liu, Y. H. Faceted and Circular Droplet Spreading on Hierarchical Superhydrophobic Surfaces. *Langmuir* **2020**, *36* (2), 534-539.
